# Friction Mechanism on Steel Surface in *n*‑Hexadecane Containing Stearic Acid Based on Cross-Sectional Observation Using Frequency-Modulation Atomic Force Microscopy

**DOI:** 10.1021/acs.langmuir.5c05564

**Published:** 2026-02-09

**Authors:** Kaisei Sato, Yuko Sato, Seiya Watanabe, Shinya Sasaki

**Affiliations:** † 26413 Tokyo University of Science, 6-3-1 Niijyuku, Katsushika-Ku, Tokyo 125-8585, Japan; ‡ Graduate School of Tokyo University of Science, 6-3-1 Niijyuku, Katsushika-Ku, Tokyo 125-8585, Japan

## Abstract

Understanding the relationship between interfacial molecular structures and their frictional properties is one of the key issues in analyzing boundary lubrication mechanisms. In this study, the interfacial structure and frictional behavior of stearic acid (SA) solution were investigated using frequency-modulation atomic force microscopy (FM-AFM) and lateral force microscopy (LFM). FM-AFM visualized two distinct repulsive regions on steel and self-assembled monolayer substrates corresponding to vertically adsorbed SA molecules and a solvation layer of *n*-hexadecane (HD) molecules oriented parallel to the surface. Interaction force analysis revealed that the upper solvation layer was disrupted under approximately 15.6 pN loading. LFM measurements demonstrated a transition in the friction coefficient near 123 pN, indicating a load-dependent change in the interfacial configuration. A comparison of FM-AFM and LFM contact pressures using the Derjaguin–Muller–Toporov model showed that variations in Young’s modulus and Poisson’s ratio had a negligible effect on the estimated contact pressure, confirming the consistency of breakthrough pressure between the two measurement methods. These findings suggest that the low-friction regime under light pressure originates from the parallel alignment of HD molecules on the vertically oriented SA film.

## Graphical abstract

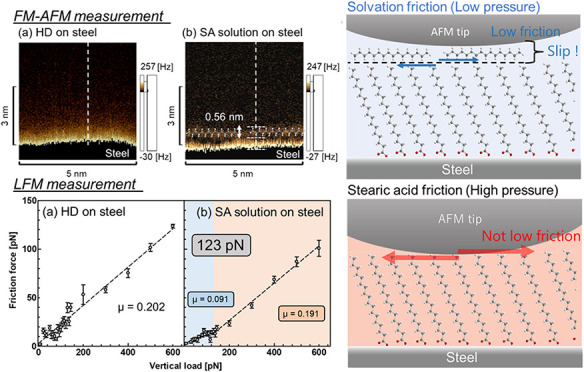

## 1 Introduction

Friction reduction directly contributes to decreasing energy loss in mechanical systems, rendering the improvement of lubricant performance crucial for addressing global environmental issues, such as energy efficiency and CO_2_ reduction. To achieve these goals, numerous studies have focused on developing lubricants and additives that effectively reduce friction at sliding interfaces, from the macroscale down to the nanoscale.^1–3^ A typical lubricant comprises a base oil and several additives, such as friction modifiers, antiwear agents, and antioxidants.^4^ Friction modifiers are essential components that directly control friction behavior under boundary lubrication, which is a complex phenomenon dominated by direct nanoscale contacts. Under such conditions, unavoidable single-asperity interactions induce interfacial wear. To better understand the nature of boundary lubrication, increasing attention has recently been directed toward elucidating the nanoscale behavior of friction modifiers under idealized single-asperity contact conditions.

Oiliness additives, as representative friction modifiers, are generally composed of molecules possessing a polar group that adsorbs onto metallic surfaces and a nonpolar hydrocarbon chain that extends outward. Traditionally, two representative adsorption models have been proposed: the monolayer model proposed by Hardy et al.^5^ and the multilayer model proposed by Allen et al.^6^ The monolayer model suggests that a single molecular layer of fatty acids can effectively separate the sliding surfaces and reduce friction. In contrast, the multilayer model assumes that thicker molecular assemblies are required to withstand applied loads. Recent experimental studies using advanced *in situ* techniques have revealed that saturated fatty acids such as stearic acid (SA) tend to form densely packed monolayers on metal or mica surfaces, supporting Hardy’s concept.^7–9^ A comprehensive review by Spikes discussed the mechanisms through which organic friction modifiers, particularly long-chain fatty acids, adsorb onto metal surfaces to form self-assembled boundary films that reduce friction at the solid–liquid interface. Ratoi et al. used ultrathin-film interferometry to measure the boundary film thickness in lubricated contacts and reported that the SA film in *n*-hexadecane (HD) was 2–3 nm thick, corresponding to a monolayer of SA.^8^ Using liquid-cell atomic force microscopy (AFM), Campen et al. found that stearic and elaidic acids generally formed 1.6 nm-thick layers that corresponded to tilted monolayers, and the SA solution in HD immediately formed a complete monolayer on mica within 3 min.^9^

In addition to the adsorbed monolayer, solvation structures—ordered layers of base-oil molecules adjacent to the adsorbed film—have been revealed by recent surface force measurements and are known to play a significant role in interfacial friction.^10–13^ Lundgren et al. reported that the confined base-oil layer above an SA monolayer exhibits a lower friction coefficient than pure HD.^11^ Similarly, Watanabe et al. used sum-frequency generation spectroscopy to reveal that the base-oil molecules arrange themselves on the SA film and reduce friction under low load.^14^ These findings suggest that both the adsorbed film and the overlying solvation layer play key roles in controlling friction.

Recent molecular dynamics (MD) simulations have revealed key insights into the molecular mechanisms of boundary lubrication by fatty acid films.^15–21^ Doig et al. found that at low SA coverage, the alkane molecules show weak ordering and no stable solvation layer forms.^15^ Conversely, Ewen et al. showed that high surface coverage results in tightly packed SA monolayers that promote layering of base oil molecules, creating an ordered solvation structure.^16^ Xu et al. demonstrated through molecular dynamics simulations that solvation layers can form even near a sharp AFM tip.^17^ They further showed that solvation layering and the associated solvation forces of alkane molecules confined near the AFM tip arise from molecular packing governed primarily by van der Waals dispersion interactions.^17^ These studies highlight that solvation layer formation is governed by van der Waals dispersion interactions, with the density and organization of the fatty acid film play a key role in determining the interfacial molecular ordering. However, the direct visualization of such solvation structures and their quantitative relationship with frictional behavior have not been sufficiently clarified, particularly on steel surfaces where practical lubricated contacts occur.

The recent advancement of frequency-modulation atomic force microscopy (FM-AFM) has enabled high-sensitivity imaging of interfacial molecular structures in liquid environments.^22,23^ Noise-reduction techniques in cantilever deflection detection systems have enabled the visualization of hydration or solvation layers near solid–liquid interfaces with subnanometer resolution. This technique has been successfully applied to study various adsorption systems, including fatty acids and phosphate esters on metallic films.^24,25^ Nevertheless, previous studies have mainly focused on model metal films prepared via sputtering, and few have addressed the influence of such solvation structures on friction on practical steel surfaces at the nanoscale.

In this study, we investigated the nanoscale lubrication mechanism governed by the combined effects of adsorption and solvation in an SA solution. The interfacial structure on the steel surface was directly visualized via FM-AFM to identify both the adsorbed SA film and the solvated HD layer. In addition, the corresponding frictional properties were quantitatively evaluated using lateral force microscopy (LFM) under controlled normal loads. By correlating the FM-AFM-derived structural forces with the load-dependent frictional transition observed in LFM using a contact mechanics model, we clarified that the low-friction regime originates from squeeze-out of the solvated HD layer rather than from the adsorbed SA monolayer. This combined approach provides an experimental basis for understanding the contribution of base-oil solvation structures to friction reduction at confined, nanoscale interfaces under single-asperity contact conditions, which represent one fundamental constituent of boundary lubrication.

## 2 Experimental Section

### 2.1 Materials

To investigate the solvation structure in an SA solution, we used a 0.1 mass% SA solution in HD for our experiments. Two types of surfaces―AISI 52100 bearing steel and a self-assembled monolayer (SAM) formed on an Au substrate―were employed to investigate molecular-level friction phenomena on single asperities. Bearing steel was selected because it is a widely used representative sliding material, allowing assessment of the relevance of the observed mechanisms to practical sliding interfaces. Meanwhile, SAM was used to visualize the structures of the solvation layers formed on surfaces covered with a monomolecular layer terminated with methyl groups. The Au substrates comprised a Cr interlayer (30 nm) and an Au overlayer of thickness 150 nm on a Si wafer. The arithmetic mean roughness (Ra) of the steel substrate was 1.1 nm, while that of the gold substrate was 0.9 nm, indicating that both substrates possessed comparable and sufficiently smooth surface conditions. The steel substrate was ultrasonically cleaned for 10 min in acetone and petroleum benzene before surface analysis. The SAM was fabricated as follows: The Au substrate was cleaned in an ozone cleaner and immersed in ethanol for 30 min. It was then immersed in a mixed solution of ethanol and 1-HD thiol (HDT) at an HDT concentration of 0.066 mol/L for 24 h to form a HDT SAM. Subsequently, the SAM substrate was immersed in ethanol for 30 min and dried prior to surface analysis. Figure 1 shows the molecular structures of the chemicals used in the experiments, HD and SA. The molecular widths of HD and SA are approximately 0.6 nm.^24^

**Figure 1:**
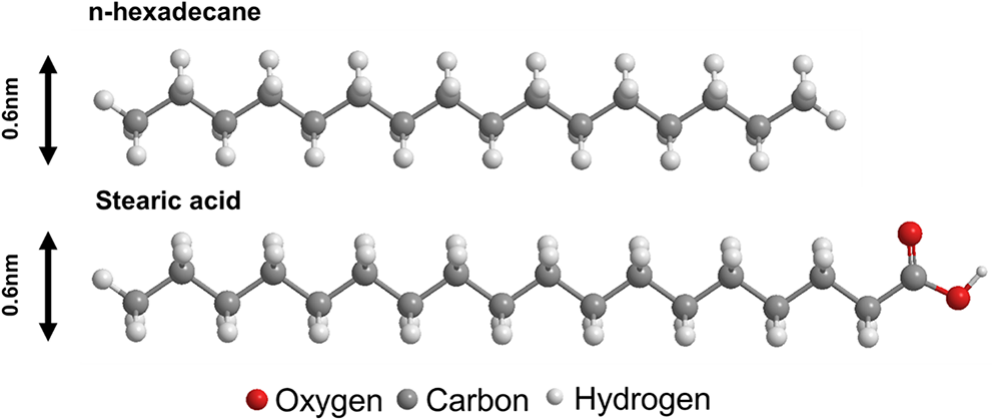
Molecular structures of HD and SA used in this study. The models show the atomic configuration of each molecule, with oxygen, carbon, and hydrogen atoms are represented in red, gray, and white, respectively.

### 2.2 AFM Measurements

In this study, two distinct AFM operation modes were employed: FM-AFM, which probes interfacial molecular structures through small-amplitude vertical oscillations of the tip, and LFM, in which a static normal load is applied while monitoring lateral forces during sliding.^26–28^ FM-AFM was used to investigate the solvation structure at the solid–liquid interface, while LFM was employed to evaluate frictional properties at the nanoscale interface under single-asperity contact conditions. All AFM measurements were performed with the tip and substrate fully immersed in the test liquid; as a result, capillary and meniscus forces are considered to be extremely small in the present study.^10,29,30^

#### 2.2.1 FM-AFM Measurements

A rectangular Si cantilever (PPP-NCHAuD, Nanosensors, Switzerland) with a tip radius of approximately 7 nm was used for FM-AFM (SPM-8100FM, SHIMADZU, Japan). It was constantly oscillated at its resonance frequency using the FM-AFM self-excited vibration system, which monitored and recorded the resulting resonance frequency shift (Δ*f*). Figure 2 shows a schematic of the FM-AFM measurements. To acquire cross-sectional images, the cantilever was operated according to the procedure shown in Figure 2(a), and the Δ*f* distributions were acquired on the ZX plane perpendicular to the substrate, as shown in Figure 2(b). In the images, brighter and darker regions correspond to positive and negative Δ*f* shifts, reflecting repulsive and attractive forces, respectively. In addition, the magnitude of Δ*f* during FM-AFM measurements is known to be influenced by the molecular density.^24^ Therefore, brighter and darker regions can also indicate areas where Δ*f* is changed owing to higher and lower molecular density, respectively.

**Figure 2:**
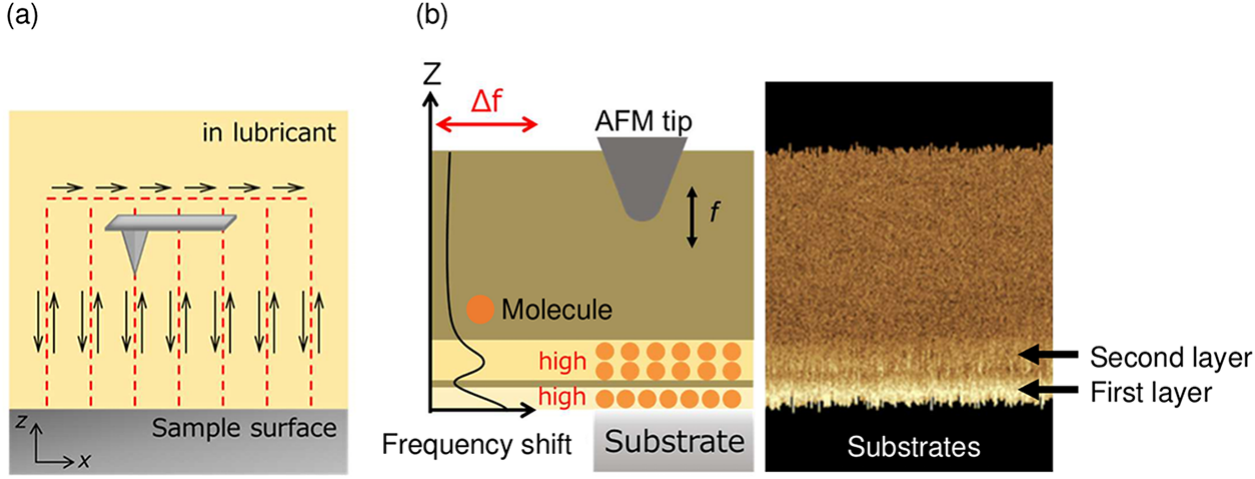
Schematic illustrations of FM-AFM-based visualization of interfacial molecular structures in lubricants. (a) Conceptual diagram showing an AFM tip interacting with confined molecular layers near a lubricated surface during scanning. (b) Schematic of FM-AFM measurements, where the frequency shift (Δ*f*) is recorded as a function of tip–sample distance (*z*), enabling detection of repulsive interaction regions corresponding to interfacial molecular layers adjacent to the substrate.

We also evaluated the interaction forces of the interfacial structure using FM-AFM. Δ*f* can be converted to the force using the equation proposed by Sader et al.^31^

1 $$F\left(z\right)=2k\int_{z}^{\infty }\left(1+\frac{A^{1/2}}{8\sqrt{\pi \left(h-z\right)}}\right)\Omega \left(h\right)-\frac{A^{3/2}}{\sqrt{2\left(h-z\right)}}\frac{d\Omega \left(h\right)}{dh}dh$$

2 $$\Omega \left(z\right)=\frac{\Delta f\left(z\right)}{f_{0}}$$

where *k*, *A*, and *f*_0_ denote the spring constant, amplitude, and resonance frequency of the cantilever, respectively. Table 1 presents the measurement parameters of the FM-AFM system used in this study. The FM-AFM measurements were conducted in 300 μL of solution at room temperature (23 °C). The normal spring constant was calculated using Sader’s method under identical conditions.^32^ In addition, for the force evaluation using FM-AFM, the standard deviation was calculated from the results of 256 force curves obtained from the ZX images.

**Table 1:** Mechanical Properties and Operating Parameters of the AFM Cantilever and Si Tip Used for FM-AFM Measurements

spring constant	N/m	18
amplitude	nm	0.6
resonance frequency	kHz	150
tip radius	nm	7
tip material	-	Si
Young’s modulus	GPa	130
Poisson’s ratio	-	0.27

#### 2.2.2 LFM Measurements

The frictional properties were investigated using AFM (Nano Navi, Hitachi High-Tech, Japan). The friction force was measured using LFM for a sliding distance of 20 nm at 20 Hz. A triangular Si-nitride cantilever (OMCL-TR400PSA; OLYMPUS, Japan) was used. The normal and lateral spring constants were calculated using the approximation equations derived by Sader et al. and Neumeister et al., respectively, as listed in Table 2.^32,33^ The sensitivity of the AFM optical system to normal deflection was calibrated from the gradient of the force–displacement (FD) curve to calculate the normal load. Friction measurements were conducted in 100 μL of solution at room temperature (23 °C). Normal loads on the order of pN were applied to preserve the solvation layers at the interface. LFM measurements were conducted with five independent measurements (*N* = 5), and the results are presented as mean values ± standard deviations.

**Table 2:** Mechanical and Material Properties of the AFM Cantilever and Tip Used in the LFM Measurements

normal spring constant	N/m	0.17
lateral spring constant	N/m	17.75
tip radius	nm	20 nm
tip material	-	Si_3_N_4_
Young’s modulus	GPa	150
Poisson’s ratio	-	0.24

## 3 Results and Discussion

### 3.1 FM-AFM Measurements

Figure 3 shows the cross-sectional Δ*f* distributions and profiles of the steel surface in the HD and SA solutions, as well as on the HDT SAM in HD. Note that the dark region below the bright area lies outside the measurement range. As shown in Figure 3(a), the repulsive force increased monotonically as the cantilever approached the steel surface in HD. This tendency indicates that HD molecules do not assemble into ordered structures on the steel surface but contribute primarily to an increase in molecular density. In contrast, Figure 3(b) indicates the presence of two repulsive-force regions, both associated with areas of elevated molecular density, on the steel surface in the SA solution. The appearance of two distinct repulsive regions suggests the existence of two molecular layers along the *Z*-axis of the steel surface in the SA solution. The polar groups of SA are generally considered to vertically adsorb onto the steel.^22^ Therefore, the bottom layer, observed as a bright region, was interpreted as the surface of vertically oriented SA molecules adsorbed onto the steel substrate. Figure 3(c) shows the Δ*f* distribution and profile on the SAM in the HD. Similar to the results for the SA solution, two repulsive regions arising from the high molecular density were observed.

**Figure 3:**
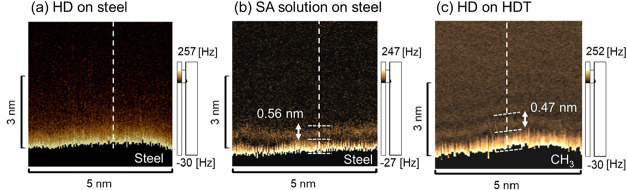
Cross-sectional frequency shift (Δ*f*) images obtained by FM-AFM for (a) HD on steel, (b) stearic acid (SA) solution on steel, and (c) HD on HDT-SAM surfaces.

Figure 4 shows the Δ*f* profile along the *Z*-axis direction obtained on the steel surface in HD and SA solutions, as well as on the HDT SAM in HD. First, the Δ*f* profile obtained on the steel surface in HD indicated that the repulsive force increased monotonically as the cantilever approached the steel surface (Figure 4(a)). Focusing on the upper region of the SA solution, the thickness estimated from the Δ*f* profile in Figure 4(b) is approximately 0.56 nm, which is comparable to the reported molecular widths of HD and SA (∼0.6 nm).^24^ When SA molecules are adsorbed onto a steel surface, their polar carboxyl groups are oriented toward the metallic substrate, resulting in an adsorbed film whose outermost surface is primarily composed of methyl groups. The polar carboxyl groups tend to segregate away from the nonpolar methyl-terminated surface. This configuration leads to the vertical orientation of SA, with methyl groups facing upward and carboxyl groups facing downward. In addition, we considered that the detected upper layer (thickness of ∼0.6 nm) corresponded to the HD and SA molecules lying flat on the SA film. Notably, the thickness of the upper repulsive region was 0.47 nm in the case of HD on HDT, which was comparable to that observed on steel in the SA solution (0.56 nm). These results confirmed that the upper layer corresponded to the HD molecules, forming a characteristic solvation structure because the measurements were performed in a pure HD system. The bottom region is attributed to the surface of the SAM, as SAMs consist of a densely packed monolayer.^34–36^ Taken together, these findings indicate that the HD molecules align parallel to the surface of the methyl-terminated SAM.

**Figure 4:**
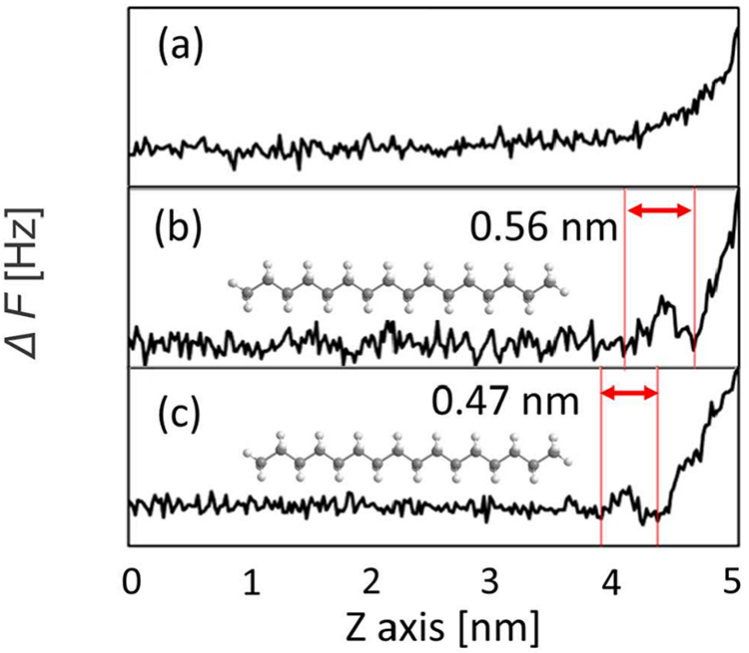
Frequency shift (Δ*f*) profiles obtained by FM-AFM for (a) HD on steel, (b) SA solution on steel, and (c) HD on HDT-SAM surfaces.

Previous molecular dynamics studies have reported that the formation of solvation layers strongly depends on the surface coverage of the adsorbed film on the substrate.^15,16^ Solvation layering at solid–liquid interfaces is known to originate from the ordering of liquid molecules driven primarily by van der Waals dispersion interactions.^17,21,30,37,38^ Earlier experimental, theoretical, and computational studies have shown that such ordering is promoted at chemically uniform, nonpolar interfaces where dispersion interactions dominate, whereas strong and spatially heterogeneous interactions at metal or oxide surfaces disturb molecular packing and suppress solvation layering.^17,21,30,37,38^ Accordingly, solvation layers were observed only when HD molecules were separated from the substrate by an adsorbed film, because the adsorbed hydrocarbon layer provided an interfacial environment in which dispersion interactions between alkyl chains could govern molecular ordering. In contrast, direct interaction between HD and the steel substrate introduced stronger surface interactions that prevented the formation of stable solvation layers. On this basis, it is inferred that stearic acid forms a densely packed adsorbed layer on the steel surface, which in turn stabilizes the solvation layers of HD molecules observed in the present study. Based on the SAM results and these considerations, an interfacial structure model is proposed for steel in an SA solution where HD molecules are arranged parallel to the SA film (Figure 5).

**Figure 5:**
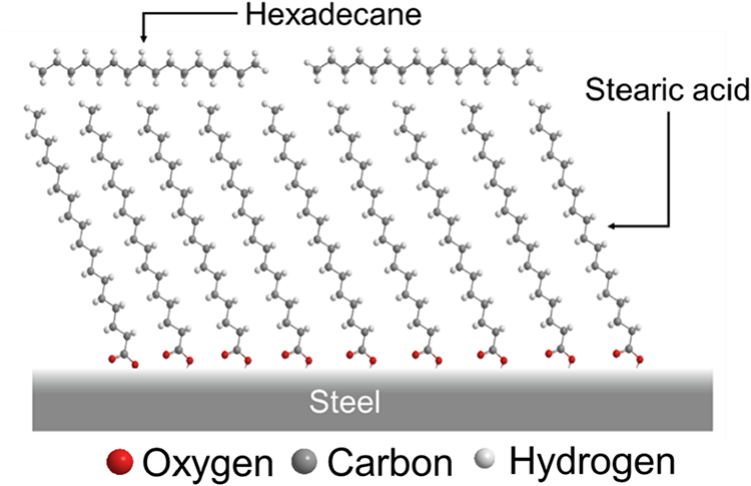
Schematic model of vertically adsorbed stearic acid (SA) molecules and solvation structure on a steel surface based on FM-AFM measurement.

We further calculated the interaction force from Δ*f* using the formula proposed by Sader et al. (eq 1). Figure 6 shows the calculated force acting on the cantilever on the steel in the SA solution. To investigate the frictional properties of the HD upper layer and SA bottom layer, we analyzed the force experienced by the cantilever as it passed through the upper layer. The force was defined as the difference between the value measured at a distance from the sample surface and the maximum force exerted at the upper layer. As shown in Figure 6, the force required to penetrate the upper layer was 15.6 ± 2.6 pN

**Figure 6:**
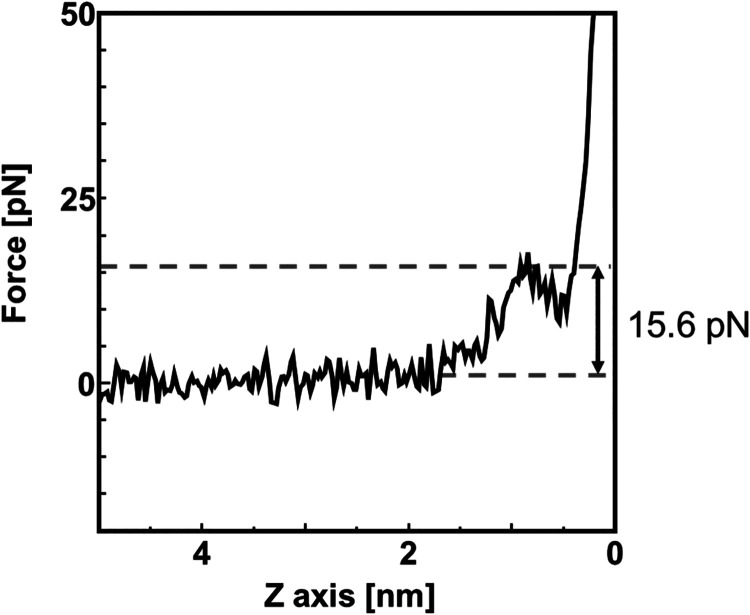
Interaction forces calculated from frequency shift (Δ*f*) using Sader equation in FM-AFM measurements dipped in SA solution.

#### 3.2 LFM Measurements

To investigate the effect of the HD arranged on the adsorbed SA film on the friction, we conducted nanoscale friction measurements at vertical loads ranging from 7.2 to 600 pN. Figure 7 shows the results of the friction measurements in the HD and SA solutions. The friction force in HD increased linearly from 7.2 to 600 pN; however, the friction force in the SA solution exhibited a superlinear increase in slope at 123 pN. In the nanoscale friction measurements, the friction coefficient was evaluated using the gradient of the friction force curve as follows^39–41^

3 $$F=\mu L+F_{0}$$

where *F* represents the friction force, μ is the friction coefficient, *L* represents the vertical load, and *F*_0_ represents the friction force at zero vertical load. Table 3 presents the friction coefficients acquired from the gradient of the friction force using the least-squares method. This result confirmed that in the SA solution, the friction coefficient clearly changed at 123 pN, and the friction coefficient from 0 to 123 pN was lower than that from 123 to 600 pN. By extrapolating the friction force to *F* = 0, the vertical load is given by $L=-\frac{F_{0}}{\mu }$ . Therefore, an effective adhesion force, *F*_ad_, can be calculated as $F_{\mathrm{ad}}=-L=\frac{F_{0}}{\mu }$ . The effective adhesion forces showed similar values in HD and SA solutions. This is likely due to the presence of HD molecules at the interface in both systems, which reduced the interaction force between the probe and the substrate surface.

**Figure 7:**
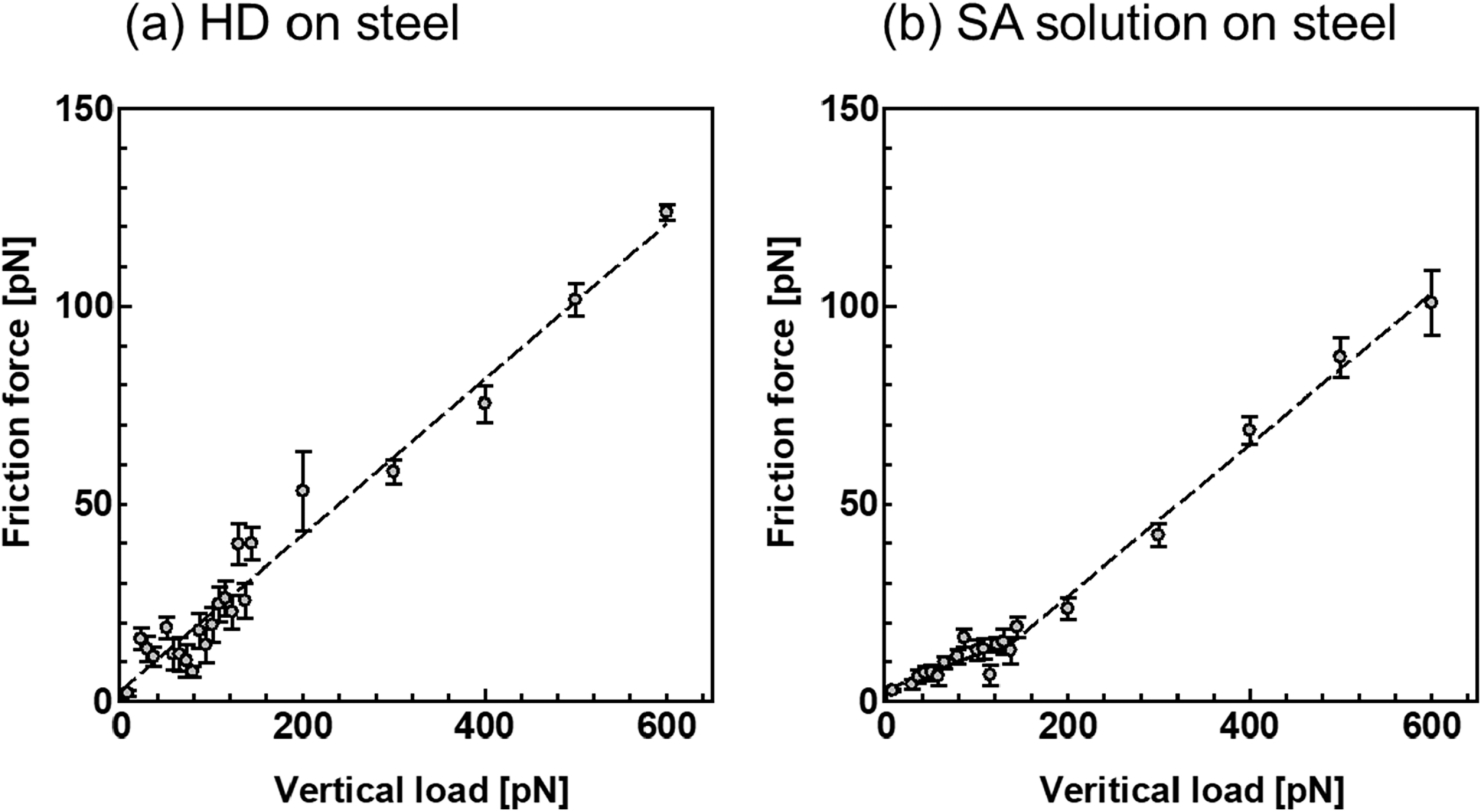
Friction force as a function of normal load for (a) HD and (b) SA solution on steel measured by LFM.

**Table 3:** Friction Coefficients in HD and SA Solutions under Low- and High-Load Regimes

friction coefficient	HD	SA solution
μ_low_ (0–123 pN)	0.202	0.095
μ_high_ (123–600 pN)	0.202	0.191

### 3.3 Estimation of Contact Pressure in FM-AFM and LFM Measurements

Under pN-order normal loads in SA solution, interfacial structural changes were observed in the FM-AFM measurements, while corresponding changes in frictional behavior were detected in the LFM measurements. Although the operating modes of FM-AFM and LFM are different, these observations are attributed to the penetration of the cantilever tip into the adsorbed film or the overlying solvation layer. Previous studies on adsorbed boundary films have indicated that the penetration of an AFM tip into the adsorbed layer is governed by the local contact pressure, with film breakdown or breakthrough occurring above a characteristic threshold pressure.^37,42–45^ To compare the threshold pressure of FM-AFM and LFM measurements, the contact pressures were calculated for both FM-AFM and LFM measurements. Several contact mechanics models have been developed to describe elastic contact at the nanoscale under different adhesion regimes.^46–53^ These models mainly differ in how adhesion forces are regarded inside and outside the contact area. The Hertz model neglects adhesion, whereas the Derjaguin–Muller–Toporov (DMT) and Johnson–Kendall–Roberts (JKR) models account for adhesive interactions in different ways, corresponding to weak and strong adhesion regimes, respectively.^50–52^ The Carpick–Ogletree–Salmeron model provides a unified description connecting the DMT and JKR limits.^53^ The appropriate contact mechanics model is commonly selected based on the Tabor parameter, which reflects the relative contributions of elastic deformation and adhesion.^46,53,54^ The estimated Tabor parameter values were much smaller than unity for both FM-AFM and LFM measurements, indicating that the present experimental conditions fell within the DMT–Hertz regime as described in the Supporting Information (Evaluation of the Contact Mechanics Model Using the Tabor Parameter). The DMT model provides a suitable framework for describing contact mechanics under weak adhesion conditions and can accommodate finite adhesive forces. In particular, despite the Tabor parameter being extremely low, an effective adhesion force on the order of several tens of piconewtons was evaluated from the LFM measurements. Accordingly, the DMT model was adopted for contact pressure estimation.

According to the DMT model, for a sphere on a flat surface, the contact radius *a* can be expressed as follows

$$a={\left\{\frac{3R\left(W+F_{\mathrm{ad}}\right)}{4E^{*}}\right\}}^{1/3}$$ 4

where *W* is the normal load, E* is the reduced elastic modulus, and *R* is the tip radius. From this equation, the mean contact pressure *p*_mean_ can be calculated

5 $$p_{\mathrm{mean}}=\frac{W+F_{\mathrm{ad}}}{\pi a^{2}}$$

The reduced elastic modulus E* is determined from the Young’s moduli and Poisson’s ratios of the sample surface and AFM tip as follows

$$\frac{1}{E^{*}}=\frac{1-\nu_{s}^{2}}{E_{s}}+\frac{1-\nu_{t}^{2}}{E_{t}}$$ 6

where *E*_s_ and *E*_t_ are the Young’s moduli of the measurement surface and AFM tip, respectively, and ν_s_ and ν_t_ are the Poisson’s ratios of the surface and AFM tip, respectively.

For the Young’s modulus of the adsorbed monolayer, Leng et al. reported values of 20–40 GPa for self-assembled monolayers based on molecular dynamics simulations.^55^ In addition, Tani et al. measured the Young’s modulus of Langmuir–Blodgett monolayers on Si substrates using Ar gas cluster ion beams and reported a value of approximately 45 GPa.^56^ In addition, the Poisson’s ratio of adsorbed molecular films is also difficult to measure directly due to experimental and methodological limitations associated with nanometer-scale thicknesses. Therefore, it is common practice in thin-film mechanics and simulation studies to assume that the Poisson’s ratio of a film is similar to that of the underlying substrate, as the substrate strongly influences the overall deformation behavior.^57,58^ Following these previous studies, the Young’s modulus and Poisson’s ratio of the adsorbed fatty acid boundary film were assumed to be 30 GPa and 0.3, respectively, for estimating the contact pressure in both FM-AFM and LFM measurements.

In the FM-AFM experiments, the solvation structure was disrupted at a normal load of approximately 15.6 pN. In the LFM measurements, a significant increase in the friction coefficient was observed at a normal load of 123 pN. Using the DMT model, the contact pressures under the normal loads in FM-AFM (normal load: 15.6 pN; adhesion force: 0 pN) and LFM (normal load: 123 pN; adhesion force: 24.2 pN (Table 4)) were calculated to be 0.235 and 0.251 GPa, respectively, indicating highly similar contact pressure levels. Furthermore, even when the assumed Young’s modulus and Poisson’s ratio were varied, the estimated contact pressures for FM-AFM and LFM remained comparable (see Supporting Information; Figures S2 and S3). This result indicated that the correspondence between the solvation-layer breakthrough observed in FM-AFM and the change in friction behavior observed in LFM is robust and does not strongly depend on the assumed elastic parameters of the surface. Furthermore, given that the estimated contact pressure was below 0.63 GPa, the influence of film transfer is expected to be minimal (see Supporting Information; Evaluation of Possible Tip Radius Change Due to Film Transfer and Wear).

**Table 4:** Fitted Parameters *F*_0_ and Effective Adhesion Forces *F*_ad_ in HD and SA Solutions

	HD	SA solution
*F*_0_	4.1 pN	2.3 pN
*F*_ad_	20.3 pN	24.2 pN

### 3.4 Nanoscale Friction Phenomenon in SA Solution

The superlinear increase in friction observed in LFM can, in principle, arise from two distinct mechanisms: (i) the shear-induced squeeze-out of a thin, low-friction solvation layer, resulting in direct sliding at a higher-friction interface, and (ii) an additional plowing contribution to friction caused by penetration of the AFM tip into the molecular film, leading to enhanced viscous or plastic dissipation within the film.^16,42,59–61^

Regarding the plowing mechanism, Salmeron reported that SAM subjected to high contact pressures on the order of gigapascals can accommodate indentation of sharp AFM tips through collective molecular tilting and the formation of molecular defects, which gives rise to enhanced frictional dissipation during sliding.^59^ Consistent with this interpretation, Gao et al. reported direct evidence of plowing behavior in molecular dynamics simulations of SA monolayers, demonstrating that tip penetration into the monolayer leads to an additional friction contribution beyond that expected from simple interfacial shear.^60^ Furthermore, Flater et al. reported that inclusion of a plowing term in analytical friction models results in a superlinear dependence of friction force on normal load, similar to that observed in LFM-based friction measurements of molecular films.^61^

With respect to the squeeze-out mechanism, Gosvami et al. reported that the last remaining HD solvation layer confined between an AFM tip and a graphene surface is squeezed out at contact pressures of approximately 0.79–0.8 GPa.^42^ In the present study, the estimated contact pressures at which the slope of the friction–load curve increases were lower than, but within the same order of magnitude as, this reported squeeze-out pressure. Based on this comparison of characteristic pressure scales, the observed change in the friction coefficient in LFM is attributed primarily to shear-induced squeeze-out of the HD solvation layer.

### 3.5 Friction Mechanism Governed by Solvation Layer

Based on the combined FM-AFM and LFM analyses, the interfacial structure and friction mechanism can be summarized as follows (see Figure 8). Under light pressure, the AFM tip slid on the solvated HD layer parallel to the SA monolayer. Shear motion occurred primarily within this molecularly ordered HD layer, and low friction was achieved during shear-induced squeeze-out of the HD layer. Once the contact pressure exceeded approximately 0.25 GPa, the solvated HD layer collapsed and direct contact between the tip and the methyl chains of the adsorbed SA film occurred, resulting in a higher friction regime. Therefore, the low-friction state under light pressures is dominated by squeeze-out of the solvated HD layer rather than by the adsorbed SA film.

**Figure 8:**
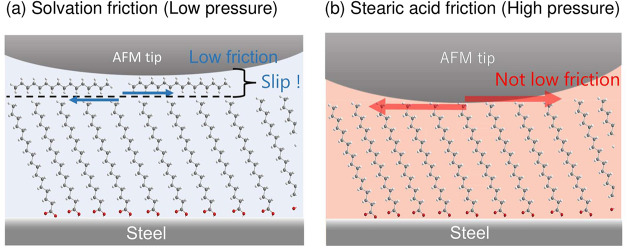
Schematic illustration of the proposed frictional mechanisms at low and high pressures for SA films in HD solution.

## 4 Conclusions

To investigate the interfacial structure on a steel surface when immersed in a lubricant, we combined FM-AFM observations with LFM-based friction measurements. The following conclusions can be drawn.

- FM-AFM cross-sectional measurements revealed two distinct repulsive regions at the steel–lubricant interface in the SA solution. The lower region was attributed to a vertically adsorbed SA monolayer, whereas the upper region corresponded to a solvated HD layer aligned parallel to the surface. Force reconstruction from the measured frequency shift indicated that the HD solvation layer was disrupted at a characteristic normal force of approximately 15.6 pN.
- LFM measurements showed a clear load-dependent transition in friction behavior in the SA solution. A low-friction regime was observed under light loads, whereas the friction coefficient increased when the load was above a critical normal load of approximately 123 pN, indicating a change in the dominant interfacial shear mechanism.
- By converting the normal loads in FM-AFM and LFM measurements into contact pressures using the DMT model, it was confirmed that the calculated contact pressures were nearly identical. This correspondence was maintained regardless of the assumed mechanical properties of the surface, such as the Young’s modulus and Poisson’s ratio.

These results demonstrate that the low-friction regime low contact pressures, in the single-asperity contact regime, originates from squeeze-out of the ordered HD solvation layer formed on the SA monolayer, rather than from the adsorbed SA film itself.

## Supplementary Material





## Notes

Confirmation of SAM film formation evaluation of the contact mechanics model using the Tabor parameter the effects of Young’s modulus and Poisson’s ratio on the contact pressure in FM-AFM and LFM measurements evaluation of possible tip radius change due to film transfer chemical structures of regents used in our experiments (PDF)
*ZX* images and *ZXY* images (MP4)

## §

Osaka University, 1–1 Machikaneyama, Toyonaka, Osaka 560–0043, Japan

## Abbreviations

AFM: atomic force microscopy
FM-AFM: frequency modulation atomic force microscopy
LFM: lateral force microscopy
SA: stearic acid
HD: hexadecane
HDT: hexadecanethiol
SAM: self-assembled monolayer
DMT: Derjaguin–Muller–Toporov
JKR: Johnson–Kendall–Roberts
