# Supplementary material for: Friction Mechanism on Steel Surface in n‑Hexadecane Containing Stearic Acid Based on Cross-Sectional Observation Using Frequency-Modulation Atomic Force Microscopy
Source: Langmuir. 2026 Feb 9;42(7):5524–32. doi: 10.1021/acs.langmuir.5c05564 (PMC12937098; doi:10.1021/acs.langmuir.5c05564)
Supplement: Supplementary file 1 [file la5c05564_si_001.pdf]

Supporting information of Friction mechanism on steel surface in n-hexadecane containing stearic acid based on cross-sectional observation using frequency modulation atomic force microscopy

Kaisei Sato [1)*], Yuko Sato [1)], Seiya Watanabe [2)**], and Shinya Sasaki [2)]

1) Tokyo University of Science, 6-3-1 Niijyuku, Katsushika-Ku, Tokyo, Japan.
2) Graduate School of Tokyo University of Science, 6-3-1 Niijyuku, Katsushika-Ku, Tokyo, Japan.

*Corresponding author: Kaisei Sato
** Current affiliation: Osaka University 1-1 Machikaneyama, Toyonaka, Osaka, 560-0043, Japan
Tel.: +81 3 5876 1334, E-mail address: kaisei_sato@rs.tus.ac.jp

Confirmation of SAM Film Formation:

The formation of the self-assembled monolayer (SAM) was verified using X-ray photoelectron spectroscopy (XPS). XPS measurements were performed before and after SAM formation, and the depth profile was analyzed by gentle Ar^+ ion etching to confirm the presence of the SAM layer. After SAM formation, a carbon signal was observed on the surface, and in addition, a distinct sulfur peak appeared in the XPS spectra, indicating the existence of sulfur-containing molecules chemically bonded to the gold substrate.

The XPS spectra of the Au 4f region exhibited two sets of doublet peaks. The first set, observed at binding energies of **83.9 eV (Au $4f_{7/2}$)** and **87.5 eV (Au $4f_{5/2}$)**, corresponds to metallic gold (Au^0).[34-36] After SAM formation, additional peaks appeared at **85.0 eV (Au–S $4f_{7/2}$)** and **89.1 eV (Au–S $4f_{5/2}$)**, which are attributed to the formation of Au–S bonds between the thiol groups of HDT and the gold surface.[34-36]

Furthermore, a sulfur signal was detected at a binding energy of **163.7 eV (Au–S 2p)**, supporting the formation of Au–S chemical bonds.[36] These results clearly indicate that the thiol groups in HDT molecules reacted with the gold surface, forming a densely packed SAM with the methyl groups oriented outward.

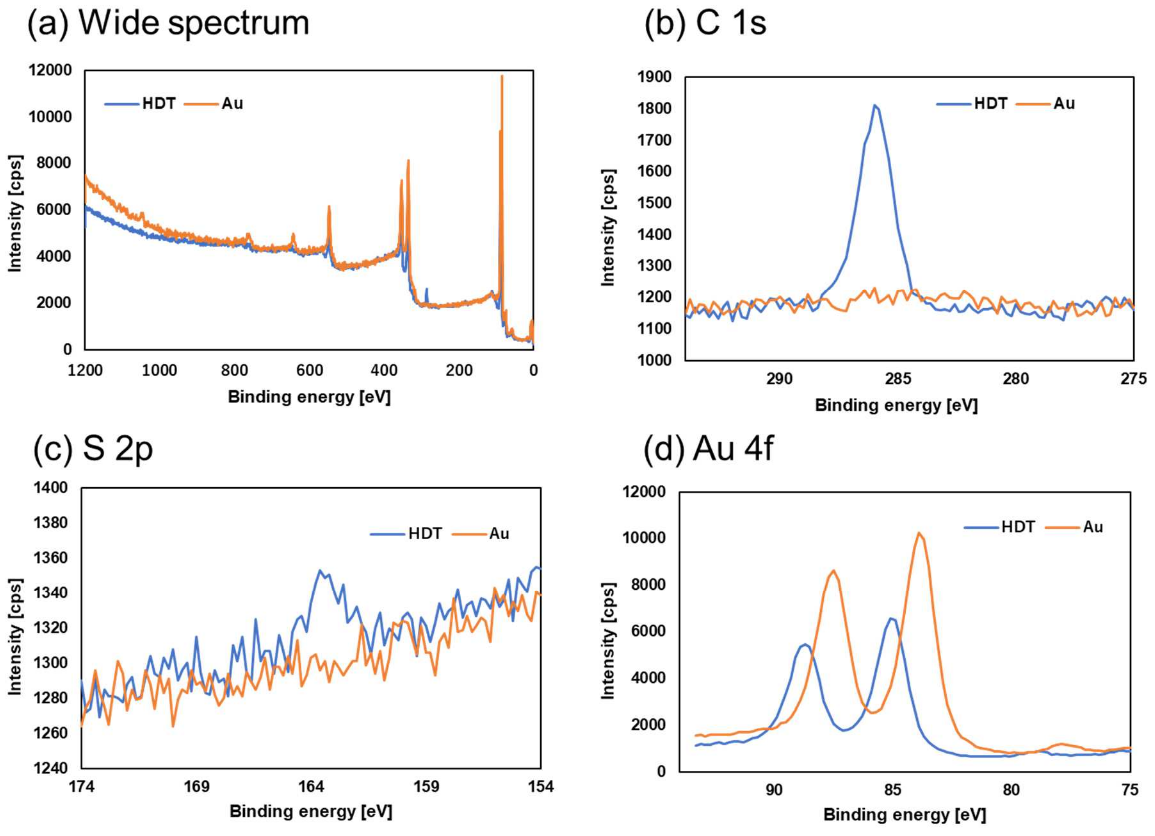

Fig. S1 XPS spectra of the gold substrate before and after SAM formation with hexadecane thiol (HDT): (a) Wide scan spectrum, (b) C 1s region, (c) S 2p region, and (d) Au 4f region.

Evaluation of the Contact Mechanics Model Using the Tabor Parameter

To assess the applicability of the contact mechanics model used in this study, the Tabor parameter (μ_t) was evaluated. The Tabor parameter provides a quantitative criterion for determining the appropriate adhesive contact mechanics model and describes the transition between the Hertz, DMT, and JKR limits.[46,53,54]

The Tabor parameter is defined as:

$$\mu_t = \left(\frac{RW_{ad}^2}{E^{*2}z_0^3}\right)^{1/3}$$

where R is the AFM tip radius, W_{ad} is the work of adhesion, E^* is the reduced Young's modulus, and z_0 is the range of adhesive interactions. The work of adhesion W_{ad} was estimated from the pull-off force measured in force–distance curve experiments. When required, the DMT relation,

$$F_{\text{pull-off}} = 2\pi RW_{ad}$$

was used. Because all experiments were conducted in a hydrocarbon liquid environment, adhesion forces were strongly suppressed, and the measured pull-off forces were extremely small, resulting in very small estimated values of W_{ad}.

Using the experimentally determined parameters, the calculated Tabor parameter was found to be much smaller than unity ($\mu \ll 1$), indicating that the present contact conditions fall within the DMT–Hertz regime. In particular, despite the Tabor parameter being extremely low, an effective adhesion force on the order of several tens of piconewtons was evaluated from the LFM measurements. Therefore, the DMT model provides a reasonable first-order approximation for estimating the contact pressure in the present nanoscale experiments. Importantly, the conclusions of this study were not sensitive to the specific choice of elastic parameters within physically reasonable ranges.

Based on these results, the validity of using the DMT model for both LFM and FM-AFM measurements was further examined. As representative values, the Young's modulus and Poisson's ratio of the substrate were assumed to be 30 GPa and 0.3, respectively, and the adhesion range z_0 was set to 0.2 nm.

For the LFM measurements, no clear adhesion force was observed in the force–distance curves; however, the effective adhesion force was calculated approximately 24.2 pN as shown in Table 4. Using this value, the corresponding Tabor parameter was estimated to be μ = 0.0050, which is well within the DMT–Hertz regime. Similarly, for the FM-AFM measurements, adhesion forces were also negligible, and the maximum negative load arising from measurement noise was approximately 2 pN. Using this value, the calculated Tabor parameter was $\mu = 0.0014$. These results confirm that the contact conditions in both LFM and FM-AFM measurements lie well within the DMT–Hertz regime.

The effects of Young's modulus and Poisson's ratio on the contact pressure in FM-AFM and LFM measurements

In the present study, different cantilevers with distinct tip radii were employed for FM-AFM and LFM measurements. Since the frictional behavior of adsorbed films and solvation layers is strongly governed by the local contact pressure, which depends on the tip geometry and the mechanical properties of the probed surface, it is essential to evaluate the influence of these parameters on the estimated contact pressure. In the main text, based on previous studies, the Young's modulus and Poisson's ratio of the measurement surface were assumed to be 30 GPa and 0.3, respectively. Using these assumed values, the contact pressures in FM-AFM and LFM were compared to verify the consistency between the two measurement techniques. However, these mechanical properties do not necessarily represent the exact values of the actual surface. Therefore, variations in the assumed Young's modulus and Poisson's ratio could potentially affect the estimated contact pressure required to break through the solvation layer in FM-AFM and LFM measurements. To assess this effect, the contact pressure was systematically calculated by varying the Young's modulus and Poisson's ratio of the measurement surface.

Figures S2 and S3 summarize the results for FM-AFM at a normal load of 15.6 pN and for LFM at a normal load of 123 pN (corresponding to a total normal load of 147 pN, given by the sum of the applied normal load and the adhesion force) using the DMT model. The left panels show the dependence of the contact pressure on Young's modulus over a wide range of values, while the right panels illustrate the effect of Poisson's ratio on the contact pressure. The results demonstrate that variations in Poisson's ratio have only a minor influence on the contact pressure in both FM-AFM and LFM measurements. In contrast, the Young's modulus significantly affects the magnitude of the contact pressure. Nevertheless, a similar trend is observed for both FM-AFM and LFM, indicating that the relative comparison between the two techniques remains valid. Even when assuming a Young's modulus of 210 GPa, which is comparable to that of typical steel materials, the estimated contact pressure is approximately 0.5–0.6 GPa. This value remains lower than the contact pressure of approximately 0.8 GPa reported by Gosvami et al. for the squeeze-out of hexadecane on graphene.[42]

These results indicate that the coincidence between the solvation-layer breakthrough pressure observed in FM-AFM and the change point in the friction coefficient measured by LFM is robust against reasonable variations in the assumed Young's modulus and Poisson's ratio. Therefore, the change in the friction coefficient observed in LFM measurements can be attributed to the squeeze-out of HD, consistent with the interpretation derived from FM-AFM measurements.

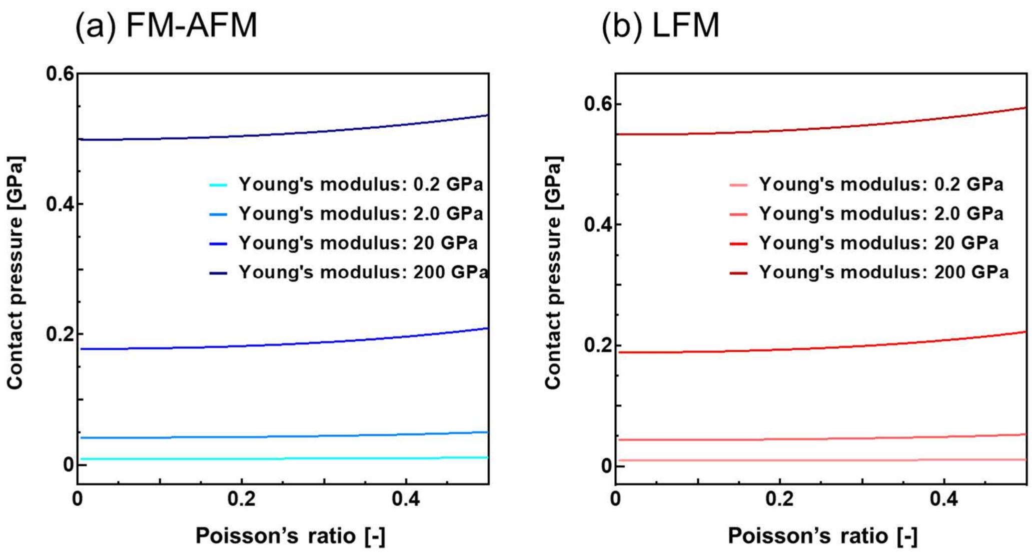

Fig. S2 Calculated contact pressure for FM-AFM and LFM measurements when changing Poisson's ratio of the measurement surface, (a) FM-AFM at a normal load of 15.6 pN (b) LFM at a normal load of 123 pN (corresponding to a total normal load of 147 pN)

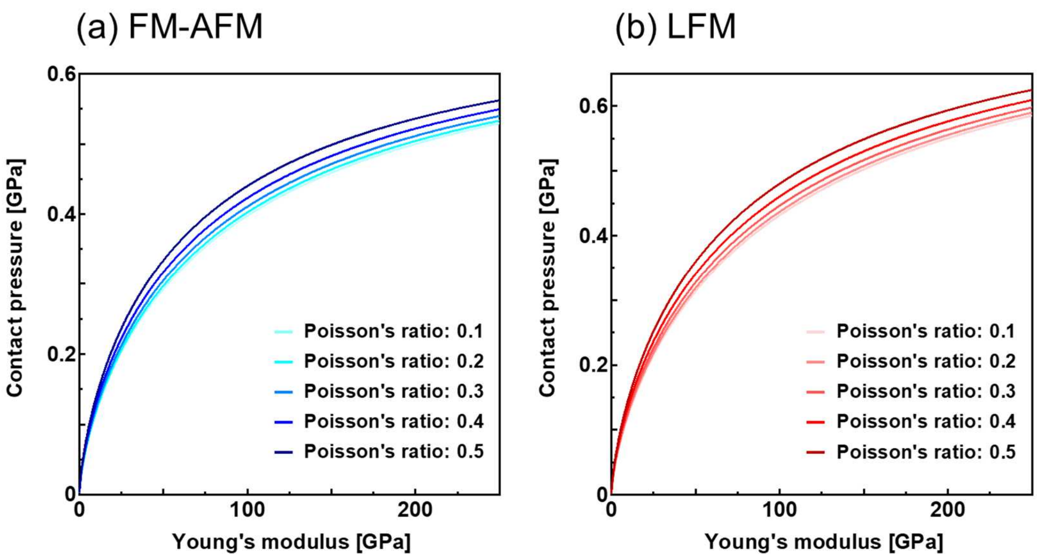

Fig. S3 Calculated contact pressure for FM-AFM and LFM measurements when changing Young's modulus of the measurement surface, (a) FM-AFM at a normal load of 15.6 pN (b) LFM at a normal load of 123 pN (corresponding to a total normal load of 147 pN)

Evaluation of Possible Tip Radius Change Due to Film Transfer and Wear

In this Supporting Information, we evaluate the possible influence of film transfer from the adsorbed stearic acid layer on the effective AFM tip radius during contact-mode LFM measurements. This evaluation is based on measurement reproducibility, contact pressure analysis, mechanical considerations of the AFM tip, and consistency between FM-AFM and LFM results.

The contact pressures in the present FM-AFM and LFM experiments were estimated to be approximately 0.25 GPa using the DMT contact model. Previous studies reporting material transfer or contamination of AFM tips during contact-mode measurements on organic or adsorbed films typically involve higher contact pressures, often in the range of several hundred megapascals to several gigapascals.[36,39,40] Therefore, the contact pressure applied in the present study is substantially lower than the pressure range where significant molecular transfer has been reported, suggesting that film transfer to the AFM tip is unlikely under the present conditions. The AFM tip used in this study was made of Si or silicon nitride (Si_3N_4). The hardness of Si is approximately 10–12 GPa, while that of Si_3N_4 is typically in the range of 15–25 GPa. These values are significantly higher than the applied contact pressure (~0.25 GPa), indicating that plastic deformation or wear of the tip apex is highly unlikely. Consequently, any change in the effective tip radius due to mechanical wear during the measurements can be considered negligible.

Table S1 Chemical structures of regents used in our experiments

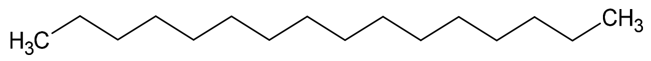
Hexadecane : $C_{16}H_{34}$
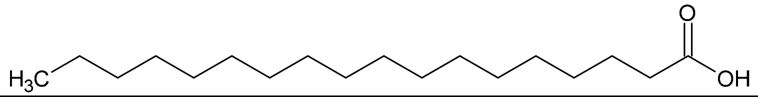
Stearic acid : $C_{18}H_{36}O_2$
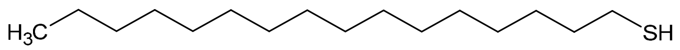
Hexadecane thiol : $CH_3(CH_2)_{15}SH$
